# Are There Any Biomarkers for Pedophilia and Sexual Child Abuse? A Review

**DOI:** 10.3389/fpsyt.2019.00940

**Published:** 2020-01-21

**Authors:** Kirsten Jordan, Tamara Sheila Nadine Wild, Peter Fromberger, Isabel Müller, Jürgen Leo Müller

**Affiliations:** ^1^ Forensic Psychiatry and Psychotherapy, Clinic of Psychiatry and Psychotherapy, University Medical Center, University of Goettingen, Goettingen, Germany; ^2^ Asklepios Forensic Psychiatric Hospital, Goettingen, Germany

**Keywords:** pedophilia, child abuse, biomarker, etiology, diagnostics, treatment evaluation, risk assessment

## Abstract

The use of biomarkers in medicine is a common and valuable approach in several clinical fields. Understanding the relationship between measurable biological processes and clinical outcomes not only is indispensable in the face of understanding physiological processes in healthy as well as in diseased organisms but also for understanding and evaluating treatment effects. Therefore, also in the context of forensic psychiatry, biomarkers and their potentially beneficial effects are of growing interest. The objective of this review is to examine if there are biomarkers that may serve as a tool to support diagnostic process, treatment evaluation, and risk assessment of pedophilic individuals and child sexual offenders. In the first part, we present an overview of the current neurobiological, as well as physiological and psychophysiological approaches to characterize pedophilia and child sexual offending. Secondly, we discuss and evaluate the impact of these approaches on the development of biomarkers for diagnosis, therapy, and risk assessment in pedophilic subjects and child sexual offenders. We conclude that a lot of research has already enhanced our neurobiological knowledge about pedophilia and child sexual offending. Although there surely exist promising parameters and approaches, in our view currently none of these is ready yet to serve as a clinically applicable diagnostic, response, or predictive biomarker for pedophilia and child sexual offending. Therefore, further work remains to be done. The development of a composite diagnostic biomarker to assess deviant sexual interest, combining several measures like functional magnetic resonance imaging, electroencephalogram, eye tracking, and behavioral approaches seems to be most promising. A valid and reliable measurement of deviant sexual interest, insensitive to manipulations could significantly support clinical diagnostic process. Similarly, regarding therapy evaluation and risk assessment, a composite biomarker to assess inhibitory control functions seems to be promising. Furthermore, the application of the Research Domain Criteria-approach, a new approach for investigating and classifying mental disorders, offers the possibility to take research to a new level.

## Biomarkers in Psychiatry

The Biomarkers Definitions Working Group of the National Institute of Health defined biomarkers as “a characteristic that is objectively measured and evaluated as an indicator of normal biological processes, pathogenic processes or pharmacological responses to a therapeutic intervention” (1, 2). Biomarkers already play a crucial role in many (bio-)medical fields as they can be used for a deeper understanding of normal, healthy physiology and the relationship between measurable biological processes and clinical outcomes (3). Therefore, the use of biomarkers opens the opportunity not only to improve the accuracy of a diagnose but can also be vital for monitoring the success of a treatment. Ideally, biomarkers should serve as clinical “tests” that diagnose the disorder or predict outcome (4). Nowadays, biomarkers do not only include molecular, blood, genetic, or neurotransmitter biomarkers but also epigenetic, and structural and functional imaging parameters. Additionally, there also exist psychophysiological, behavioral, and digital behavioral biomarkers, such as neural patterns associated with working memory, which are disrupted in patients with schizophrenia (5, 6), eye tracking measures of social attention as an indicator for autism (7), behavioral movement biomarkers to predict later motor and cognitive dysfunctions in young high-risk infants (8), or physical activity assessed with mobile technologies (9).

Before any biomarker can be used in medical settings, it needs to be validated in large-scale clinical trials. Biomarkers only are valid if a number of preconditions are satisfied: i) there must exist a statistically significant relationship between the biomarker and a defined clinical endpoint, ii) the biomarker and the defined clinical endpoint must be causally or mechanistically related, iii) sensitivity and specificity must be sufficient to distinguish between false positives, false negatives, true negatives, and true positives, and iv) the method should be reproducible across all clinically relevant fields (4). In addition, other authors recommend that biomarkers should preferably be minimally invasive, and acceptable to the patient (10).

Prata et al. (4) simplified a sophisticated scheme to evaluate biomarkers proposed by Lassere (11) and adapted it for use in the psychiatric field; a two-dimensional scale assessing quality of evidence (similarly to phase I–IV of clinical drug trials) and effect size. Thus, a biomarker is considered to be validated if it shows a positive result in a study (*p* < .05, corrected for multiple comparisons). Furthermore, this study should i) demonstrate large effect sizes, ii) be controlled for relevant extraneous variables, iii) be performed with an explicit *a priori* intent to discover a precisely defined biomarker, and with adequate power informed by previous positive studies of the same biomarker (i.e., replication in at least two studies in a larger cohort) (4). However, even valid, applicable biomarkers are not useful until shown to provide a meaningful advantage when incorporated into decision-making or clinical care (1, 4).

In contrast to other medical disciplines, the development of biomarkers is a particular challenge for the field of psychiatry, as diagnoses are solely based on a descriptive collection of behaviors without the availability of any objective test to stratify patients (i.e., DSM/ICD) (5). The use of biomarkers in psychiatry provides an opportunity to enrich the subjective descriptive classification with objective and tangible measures in order to improve diagnosis, treatment, and prognosis. Since the 1980's, a large amount of research has led to remarkable progress in biological research and a critical insight into the pathogenesis of various psychiatric disorders, considering also associations between genes, brain, and social behavior [e.g. (12) for an overview see: (13)]. However, as pointed out by several authors, clinically translatable biomarkers in psychiatry are yet to be identified [e.g. (12, 14)]. In a large systematic and quantitative review, Prata and colleagues analyzed over 3,200 articles investigating psychosis-related biomarkers (4). Out of these, fewer than 200 studies investigated biomarkers longitudinally, and assessed their predictive utility with regard to course of disease and treatment response. According to the evaluation scheme developed by Prata and colleagues (see above), only one biomarker passed the *a priori* threshold for clinical applicability (4, 15). Venkatasubramanian & Keshavan identified some possible reasons for this failure: i) as mentioned above, current classificatory and diagnostic systems in psychiatry are primarily symptom-based, ii) methodological limits of the existing studies on biological abnormalities in psychiatry, iii) lack of valid “*in-vitro*” models for psychiatric disorders, iv) issues related to conceptualizations of pathogenetic paradigms for psychiatric disorders (14). Due to the complexity of psychiatric disorders, Lozupone et al. claim not to use single biomarkers, but a combination of diverse biomarker types (12). This could lead to an improvement of diagnosis, treatment, and prognosis of psychiatric patients on a personalized level.

As compared to general psychiatry, the field of forensic psychiatry is rather small. Nevertheless, a substantial amount of research has been conducted in order to identify biological underpinnings of forensically relevant disorders. In accordance with this research, the Integrated Theory of Sexual Offending (ITSO), as one theoretical approach to explain sexual offending, provides a framework including an interaction between neurobiological and ecological factors (16). With respect to pedophilia, Tenbergen et al. proposed a conceptual neurodevelopmental framework, with pedophilia as a complex and multifactorial phenomenon, in which the influences of genetics, stressful life events, specific learning processes, as well as structural brain changes may generate the specific phenotype of child sexual preference (17). In our review, however, we will solely concentrate on biological parameters. Obviously, we are aware of the significance of other, highly relevant factors, e.g., environmental, parental, social, and cultural factors. Nevertheless, those factors are outside the scope of this review. Furthermore, due to the low prevalence of pedophilia in women and the low level knowledge regarding pedophilic women (18, 19), we will only discuss research focusing on men.

At this point we would like to emphasize that obviously research on biomarkers has a different aim than research on the pathoetiology of pedophilia. Studies focusing on biomarkers are interested in biological markers, which could classify between groups, e.g., for diagnostic purpose. In contrast, studies interested in the underlying neurobiological pathoetiology of pedophilia want to understand the causes of pedophilia or at least underlying neurobiological processes. Currently, there are no studies explicitly intending to develop biomarkers for pedophilia and child sexual abuse. One exception might be approaches aiming to classify subjects with respect to their sexual interest in order to support the diagnostic process. Therefore, in the following, we will first summarize the current neurobiological, as well as physiological and psychophysiological approaches to characterize pedophilia and child sexual offending. Secondly, we discuss and evaluate the impact of these approaches on the development of biomarkers for diagnosis, therapy, and risk assessment in pedophilic subjects and child sexual offenders. If available, criteria of psychometric quality, such as discrimination accuracy and validity are described.

## Biological Underpinnings of Pedophilia and Child Sexual Offending—The Current Knowledge

Sexual child abuse is one of the most destructive events for child development. On behalf of the victims of child sexual abuse and for the general public, research on the underpinnings of pedophilia and child sexual offending is of great importance. In the public in general, pedophilia and child sexual offending are often used synonymously. However, pedophilia neither is a necessary nor a sufficient precondition for child sexual abuse. According to Seto (18) about half of child sexual offenders fulfill diagnostic criteria of pedophilia (20).

Pedophilia belongs to disorders of sexual preference (ICD-10), and paraphilic disorders (DSM-5) respectively. According to the ICD-10 (21) “pedophilia” (F65.4) is defined as a “persistent or dominating sexual preference for prepubescent children” with a duration of at least 6 months. A person being diagnosed with pedophilia has to be at least 16 years old and at least 5 years older than the child/the children. Furthermore, the person has to act upon his sexual preference or is suffering from his deviant sexual preference. The DSM-5-criteria of the so called “pedophilic disorder” (302.2) are very similar, namely “recurrent, intense sexually arousing fantasies, sexual urges, or behaviors involving sexual activity with prepubescent child or children over a period of at least 6 months, which causes marked distress or interpersonal difficulty or the individual has acted on these sexual urges.” However, “if persons report an absence of feelings of guilt, shame, or anxiety about these impulses and are not functionally limited by their paraphilic impulses, and their self-reported and legally recorded histories indicate that they have never acted on their impulses, then these individuals have a pedophilic sexual orientation but not a pedophilic disorder” (22). Currently, most published (neurobiological) research does not differentiate between pedophilic disorder and pedophilic sexual orientation according to DSM-5. Furthermore, some studies did not apply diagnostic criteria as defined in ICD-10 or DSM-4/DSM-5 due to unavailability of data, while others used different kinds of criteria and methods for grouping (e.g., victim age, responses in penile plethysmography). Therefore, to enhance readability, in the following, the term “pedophilia” will be used for subjects with a sexual orientation toward children, independently of the diagnostic system or method that was used. If available, details will be given about offense status.

In recent years, some research groups have focused on the differentiation between pedophilia and child sexual offending. Interestingly, as will be shown below, some neurobiological parameters seem to belong to pedophilia, while others seem to be associated with sexual offending. Accordingly, it can be assumed that biomarkers for pedophilic offenders differ from biomarkers for pedophilic non-offenders and non-pedophilic offenders. Hence, if possible, we describe these differentiations.

### Genetic and Prenatal Factors

#### Genetic Factors

Brain development is a complex organization of processes under genetic, epigenetic, hormonal, environmental, and immune regulation, and consequently is vulnerable to a variety of disturbances (23). Pre- and perinatal factors are therefore, among others, important predictors of many later life outcomes, including but not limited to criminality and psychopathology. Also for pedophilia, a neurodevelopmental basis has been suggested [e.g., (17)]. Even though several biological parameters supporting this hypothesis have been identified, more research is needed to strengthen existing empirical evidence and to clarify specificity. In a group of healthy male twins and their siblings (*N* = 3,967), Alanko et al., for instance, found a small amount of variance (14.6%) attributable to nonadditive genetic influences (heritability) for self-reported sexual interest in children (24). Later, the same group reported small genetic effects for male pedophilia: several SNPs (single-nucleotide polymorphisms) linked to androgen, estrogen, prolactin, corticotropin, serotonin, and oxytocin were associated with self-reported sexual interest in children in a community sample, but only before controlling for multiple testing (25). Examining paraphilic sexual offenders and non-offending controls, Jakubczyk et al. failed to find an association between a history of a sexual offense and the distribution of genotypes or alleles in several analyzed polymorphisms, linked to dopamine, serotonin, monoamine oxidase A, and the brain-derived neurotrophic factor (26).

#### Minor Physical Anomalies and Congenital Malformations

Minor physical anomalies (MPAs), superficial deviations from typical morphological development, such as malformed ears or toes, or fine electric hair, develop prenatally. MPAs might be external markers of abnormal brain development, as both the central nervous system and the skin derived from the same ectodermal tissue *in utero*. They are supposed to develop during the first and/or early second trimester of gestation (27–29). Even though the exact mechanism remains elusive, there are some indications that besides genetic factors, also environmental perturbations (e.g., hypoxic events) during early embryonic period may determine the extent and nature of malformations (27, 30). Research supports the hypothesis of an association between MPAs and several neuropsychiatric disorders, such as autism and schizophrenia (31, 32). Pedophilic individuals seem to exhibit a greater number of MPAs relative to samples of individuals with schizophrenia as well as healthy controls: Dyshniku and colleagues found that MPA indices were positively associated with multiple indicators of pedophilia, including phallometric responses to sexual stimuli, number of child but not adult victims, and possession of child sexual exploitation material (30). There is also a relationship between adult men's height and pre- and perinatal factors, such as genetic predisposition or conditions *in utero* (33). Fazio and colleagues found a reduced measured height and reduced leg length in pedophiles as compared with teleiophiles. The magnitude of this difference was similar to the difference found in other biologically based neurodevelopmental disorders (33). Using a large data-set from Swedish population-based registers (13,773 sex offenders, 135,953 violent non-sexual offenders, 680,120 matched controls), Babchishin and colleagues found that two perinatal factors, being small for gestational age, and a small head circumference were both associated with risk of sexual, and non-sexual violent offenses (adjusted odds ratios between 1.12 and 1.51) (34). Furthermore, any congenital malformation (according to ICD10: P00-P99) had a small effect on sexual offending against children [adjusted odds-ratio: 1.15, *CI* (1.02–1.30)], but not against adults. However, this association reached level of significance also for non-sexual violent offenses [adjusted odds ratio: 0.91, *CI* (0.88–0.94)] (34), which raises the question of whether those parameters are rather biomarkers for violent offending than for child sexual offending or pedophilia. In a subsequent study, Babchishin et al. compared a subset of 655 child sexual exploitation material offenders (CSEM), individuals who had additionally conducted contact sexual offenses against children, as well as with 3,928 matched controls (35). Any congenital malformation remained the only pregnancy-related independent risk marker for mixed offenders [CSEM and contact offenses, adjusted odds ratio: 1.7, *CI* (1.2–2.4)], but not for CSEM-exclusive offenders (35).

#### Handedness and Androgens

There is evidence that the fetus exhibits lateralized behavior from as early as 10 weeks of gestation, as soon as it independently moves its arms. This can be seen as a precursor of lateralized postnatal behavior (36). In the general population, about 90% are right-handed. Left- and mixed handedness has been reported in up to 60% of individuals suffering from several neuropsychiatric and developmental disorders, such as autism spectrum disorders, attention deficit hyperactivity disorder, schizophrenia, and different forms of addiction (37). Research has further linked left- and mixed handedness to deviant sexual interest, pedophilia, and sexual offending against children, supporting the view of a neurodevelopmental origin of paraphilic sexual preference (38). Analyzing a large sample (*N* = 1,857), Fazio et al., for instance, described higher rates of non-right handedness (sinistrality) in pedophilic sex offenders (14.6%), and increased ambiguous handedness in offenders with a sexual preference for pubescent children (12.4%) compared to teleiophilic offenders [7.4% resp. 8.8%; *χ^2^*(1, *N* = 1,712) = 13.62, *p* < .001]. Interestingly, the laterality quotient also was significantly associated with the subjects' pedophilic phallometric index score (39).

Considering the ontogenesis of handedness, recent research on molecular epigenetic mechanisms suggests that instead of single genetic factors, particular asymmetries in DNA methylation might affect asymmetric gene expression in the brain that, in turn, mediates handedness (40). In line with this, Schmitz and colleagues showed that birth stress (e.g., premature birth, breathing difficulty at birth) might be a factor that affects DNA-methylation in the promotor region of the NEUROD6 candidate gene, which could be associated with left-handedness (40). NEUROD6 is leftward asymmetrically expressed in the fetal brain, and acts as a differentiation factor for neural precursor cells in the developing brain. The absence of NEUROD6 is associated with reduced glutamatergic network activity and disruption of neocortical projections of the corpus callosum and other commissural fibers (40–42).

Regarding sexual preference, hormonal factors, such as prenatal testosterone levels, are of special interest. The earliest known effects of androgens during the prenatal phase, the so called “organizational effects,” do not only lead to sexual differentiation of the periphery in mammals but also to sexually dimorphic brains, with testosterone exposure resulting in male-typical development (masculinization), and the relative absence of testosterone resulting in female-typical development (feminization) (43–45). Interestingly, testosterone and its androgen receptor (AR) do not appear to be directly responsible for the perinatal masculinization of the brain. It is rather the local aromatase-dependent conversion of testosterone to estradiol that accounts for these effects (46, 47). The ratio between the second and the fourth digit (2D:4D ratio) has been considered as a potential marker for prenatal testosterone exposure. However, due to ethical reasons, prenatal testosterone levels cannot be manipulated in humans and causal relationships cannot be derived. Although it is still being debated, research indicates a negative association of the 2D:4D ratio and prenatal testosterone levels. In other words, a lower 2D:4D might indicate a higher prenatal testosterone (stronger masculinization). On average, the 2D:4D is smaller in males than in females [for an overview see: (48)]. A higher 2D:4D ratio in the left compared to the right hand might be associated with a tendency toward left-handedness, but this effect was not consistently reported (37). In a community sample of 200 heterosexual men, for instance, Rahman et al. found that men with stronger paraphilic sexual interests had a higher right-hand 2D:4D ratio and a trend for a lower Edinburgh Handedness Inventory scores (i.e., less right-handed) compared to men with lower paraphilic sexual interests (49).

Regarding underlying neurobiological mechanisms, not only classical genomic actions at the transcription level might play a role (modulating synapse growth, changing neurotransmitter production etc.), but also the receptor binding capacity of the AR. A particular polymorphism on the AR gene, the CAG (cytosine-adenine-guanine) repeat polymorphism is of functional importance, with shorter CAG repeats being related to a greater expression of AR protein (i.e., larger number of AR receptors) and increased transcriptional activity of AR. As a result, men with shorter CAG repeats appear to convert the same concentration of androgen into larger physiological effects than do men with longer repeats [for an overview see: (43, 50, 51)]. Although there does not seem to be a clear correlation between violent criminal activity and AR repeat polymorphism, some studies found that violent criminals, rapists, and murderers had shorter CAG repeat length than non-violent controls (52, 53).

In a recent study with 194 subjects, 2D:4D ratios were inversely associated with the total number of child sexual offenses, but not with pedophilia itself, supporting the role of prenatal testosterone in the development of delinquent behavior (54). The authors did not find a main effect for the CAG repeat length, but in the non-offending group, shorter CAG repeats were linked with higher percentage of AR-receptor methylation. They interpreted this as an indicator for a regulatory mechanism to maintain normal functioning system, i.e., the higher the AR receptor functionality (shorter CAG repeats), the less ARs are synthesized (high methylation level). Most interestingly, this regulatory mechanism was not seen in the offending population (independently of diagnosis of pedophilia), which was interpreted as an impaired regulatory mechanism between genetic and epigenetic factors in the androgen system (54). Unfortunately, the authors did not report any data about handedness. The question of whether or not there is an association between handedness and the length of CAG-repeats was nevertheless examined by another research group. Analyzing a large sample of healthy adults (*N* = 1,057), Arning et al. found that longer CAG-repeats were related to a higher incidence of non-right handedness (i.e., less lateralization). As mentioned above, longer CAG-repeats are linked to less efficient AR-function. The authors therefore concluded that differences in AR-functioning in the developing brain could be one of the factors that determine individual differences in brain lateralization (55).

### Structural and Functional Imaging

Magnetic resonance imaging and electrophysiological studies constitute another interesting biological source of information to characterize pedophilia and child sexual offending.

#### Structural Imaging

So far, three neurobiological theories for pedophilia have been proposed (50, 56). The frontal-dysexecutive theory was put forward by Graber et al. (57) and assumes that structural and functional damage to the frontal lobe might lead to behavioral disinhibition, which favors pedophilic behavior. The temporal-limbic theory, on the other hand, posits that the temporal and limbic brain regions play a major role in sexual functions. Especially, lesions of the temporal lobe are associated with hypersexual behavior (58). The dual-dysfunctional theory connects the former theories, assuming dysfunctions in both temporal and frontal brain areas. According to the latter theory, hypersexual behavior caused by temporal deficits—together with behavioral disinhibition caused by frontal deficits—leads to pedophilic behavior. However, these theories might explain hypersexual and disinhibited behavior, but not a pedophilic sexual interest itself. Furthermore, hypersexual and disinhibited behavior could also be related with other mental disorders, such as other paraphilias/paraphilic disorders, hypersexuality itself, and neurodegenerative disorders (59).

Regarding pedophilia, early pioneer studies found volume reductions in sexually relevant brain regions, such as the amygdala, hypothalamus, limbic gyri, orbitofrontal cortex, ventral striatum, and insula in pedophilic compared to non-pedophilic subjects [e.g., (56, 60, 61)]. However, these earlier studies did not distinguish between pedophilia and child sexual abuse. Recent reviews and meta-analyses investigating differences between individuals sexually oriented toward children and individuals sexually oriented toward adults revealed that their brain structures are very similar in nature (17, 62, 63). As indicated by two recent studies, aberrant neuroanatomy might rather be associated with child sexual abuse than with pedophilia (64, 65). Applying voxel based morphometry (VBM) in 219 individuals, Schiffer et al. failed to find group differences in the relative gray matter volume specifically associated with pedophilia (64). However, non-offending pedophiles (*n* = 60) exhibited larger gray matter volume in the right temporal pole than offending pedophiles (*n* = 58) or non-pedophilic controls without any history of child sexual offending (*n* = 101). The gray matter volume in the right temporal pole was negatively associated with self-focused sexual behavior in offending pedophiles but also in healthy controls. Interestingly, the risk of re-offending [assessed with the SSPI-2, Screening Scale of Pedophilic Interest 2^nd^ version, (66)] was associated with lower local gray matter volume in the right dorsomedial prefrontal cortex/anterior cingulate cortex. This finding might be related to research results on deficient inhibitory control in offending pedophiles (see below) (64). In a large sample of 283 individuals, Lett et al. could further demonstrate with moderate effect sizes that as compared to non-offending pedophiles (*n* = 77) and healthy controls (*n* = 133), pedophiles with child sexual offenses (*n* = 73) showed lower IQ-performance, reduced cortical thickness in the right motor cortex, reduced cortical surface area comprising bilateral frontal, temporal, cingulate, and insular regions, and reduced white matter fractal anisotropy particularly in the corpus callosum (65).

#### Functional Imaging—Functional Magnetic Resonance Imaging

Similarly, functional imaging studies examining neural underpinnings of child sexual offending revealed that a diminished fronto-limbic functional connectivity at resting state is rather linked to child sexual offending than to pedophilia (67, 68). According to the authors, these findings may support the theory of disturbed fronto-limbic functioning as a possible causal factor for child sexual offending regardless of pedophilia. Interestingly, a reduced fronto-limbic functional connectivity (as a response to provocations) has also been shown for violent and psychopathic offenders (69, 70). From studies with healthy subjects, it is known that there is a strong top-down inhibitory control of prefrontal over limbic structures (especially amygdala), mediating responses to provocations [for review see: (71)]. Hence, a diminished fronto-limbic functional connectivity could be seen as a neurobiological correlate for an (general) impaired ability to assert inhibitory control on behavior.

Another large amount of functional imaging research has been conducted in order to understand the neural underpinnings of child sexual interest, and to develop an “objective” instrument, a biomarker, which could support the diagnostic process of identifying pedophilia itself. A deviant sexual preference is one of the strongest single predictors for sexual-offense recidivism (72). Currently, the Western European standard for the assessment of sexual interest are self-reports. However, it is known that self-reports and questionnaires are susceptible to denial or faking (73), which is of special relevance in the forensic context. Avoiding this problem, functional imaging approaches use hemodynamic brain responses to sexual stimuli to gather information about the participant. This idea is based on the assumption that sexually relevant features of stimuli are preferentially processed, i.e., preattentively selected, and automatically trigger focal attention to these sexual aspects (74). These attentional processes can be measured at behavioral but also at neural level. With the four-component model of sexual arousal, Redoute and colleagues proposed four excitatory (cognitive, motivational, emotional, autonomic) and one inhibitory component to describe the processing of sexual stimuli at behavioral and neural level (75, 76). Early pioneer studies reported differences in several sexually relevant brain regions when comparing pedophilic child sexual offenders and healthy subjects [e.g., (77–79) ]. Recent studies yielded evidence that pedophilic subjects exhibit similar brain activations as healthy subjects while watching their preferred sexual stimulus, indicating that the four-component model of sexual arousal does not only hold true for healthy individuals but also for individuals with a sexual preference for children [for review see: (17, 62, 63, 76, 80)]. Supporting these results, Polisois-Keating and colleagues did not find significant differences between the groups in a meta-analysis with 123 subjects (80). Current research also confirms this view, and adds the idea that the right inferior temporal gyrus might be a possible candidate region mediating sexual arousal in patients with pedophilic disorder (81). Comparing responses in pedophilic outpatients (*n* = 15) and healthy controls (*n* = 15) to the presentation of sexually preferred and non-preferred stimuli, two areas in the right temporal gyrus showed increased (BA37) and decreased responses (BA20) in pedophiles. According to the authors, these areas could play an opposite role, i.e., an activating and inhibiting role in sexual arousal (81).

The typical experimental designs in this type of research, i.e., passive viewing of sexual stimuli, are, however, susceptible to manipulation. As a consequence, researchers have started to develop alternative means for the assessment of sexual interest. For instance, Jordan and colleagues have used an active cognitive task to capture healthy subjects' attention while they were simultaneously presented with task-irrelevant sexual stimuli. Results indicated that such tasks can indeed be used to measure sexual interest in healthy subjects (*N* = 22) (82). Another stimulation design, useful for avoiding possible manipulations by the subjects, seems to be the subliminal presentation of visual sexual stimuli (83, 84). Subliminal stimuli are shown with a presentation time of 50 ms at the most. The threshold of 50 ms ensures that the stimuli are, in most instances, not consciously perceived by the subjects, which decreases the possibility of manipulation of the subjective reaction (85). Applying a promising automatic pattern classification algorithm of brain responses, Ponseti and colleagues demonstrated that pedophilic out-patients (*n* = 24, 50% with sexual offenses) and non-pedophilic healthy controls (*n* = 32) could be classified with high sensitivity and specificity according to their responses not only to supraliminally presented, preferred, and non-preferred sexual stimuli (sensitivity 88%, specificity 100%) but also to face stimuli (sensitivity 95%, specificity 91%) (86, 87).

In a large sample (*N* = 104), Cantor et al. (88) further demonstrated a wide-ranging increased functional connectivity in the default mode network, with regional increases and decreases in the frontoparietal network in pedophilic sex offenders (*n* = 37) compared to non-sexual offenders (*n* = 28) and non-offenders (*n* = 39). Interestingly, most of these regions are known to respond to sexually relevant stimuli, again supporting the four-component model of sexual arousal for pedophiles (88). Based also on previous studies, which showed differences in structural connectivity (voxel based morphometry, diffusion tensor imaging), Cantor et al. proposed a pattern of *dys*connectivity rather than *dis*connectivity as a neuroanatomic substrate of pedophilia (56, 88, 89).

Functional imaging has not only been applied to assess sexual interest, but also to characterize cognitive aspects in pedophilic subjects and sexual child offenders, such as inhibitory control abilities. As one of the first research groups, Habermeyer et al. (90) examined a small sample of pedophilic sex offenders (*n* = 11) applying a go/no-go task to measure response inhibition behavior. In comparison to non-offending healthy controls (*n* = 7), pedophilic offenders exhibited slower reaction times and a less accurate visual target discrimination, which was accompanied by attenuated deactivation of brain areas belonging to the default mode network. Based on these results, Habermeyer et al. assumed a higher degree of inattention and increased self-referential processes in their pedophilic sex offenders while performing the no-go task (90). Applying a similar task with larger groups, Kärgel et al. (91) could differentiate offending (*n* = 40) and non-offending pedophiles (*n* = 37). While no general group differences were found between pedophilic subjects and non-offending healthy controls (*n* = 40), non-offending pedophiles exhibited superior inhibitory control behavior compared to offending pedophiles. Furthermore, an increased inhibition related activity in left posterior cingulate and left superior frontal cortex, areas associated with effective cognitive functioning, was found in non-offending pedophiles. According to Kärgel et al., these data indicate a better inhibitory control in pedophiles who successfully avoid committing hands-on sexual offenses against children (91).

Supporting functional magnetic resonance imaging (fMRI) and EEG studies (see below), neuropsychological research has shown executive dysfunctions in child sexual offenders (92). Interestingly, a recent neuropsychological study supports the results by Kärgel et al. (91) in so far as executive dysfunctions (worsened response inhibition abilities) are related to offense status rather than pedophilic preference (93).

#### Functional Imaging—Electroencephalogram

In contrast to fMRI, the application of electroencephalography (EEG) stands out for its high temporal resolution (1 ms) and therefore allows for the detection of very early and fast brain responses to perceived stimuli. To our knowledge, besides a few older studies [e.g., (94)], only two recent studies have investigated the application of EEG in pedophilic individuals and child sexual offenders. Knott and colleagues (95) used event-related brain potentials (ERP) to investigate the time course of the explicit processing of adult erotic, emotional, and neutral pictures in 22 pedophilic sex offenders and 22 healthy controls. In general, the ERPs elicited by emotional stimuli were similar in pedophilic sex offenders and controls, but an early positive component (P2) was significantly attenuated and slower in pedophilic individuals compared to controls. The authors interpreted the results in terms of a failure of rapid attentional capture by adult erotic stimuli, which could reflect the relatively diminished sexual interest in adults (95). Based on the knowledge of impaired response inhibition in child sexual offenders, Rosburg et al. (96) investigated these processes by means of a go/nogo task in a sample of 21 pedophilic contact child sexual offenders (CSOs), 19 non-contact CSOs (child sexual exploitation material offenders, pedophilic status not accessed), and 21 healthy controls. Results revealed that response inhibition, processing of stop-signals, and error detection were not necessarily impaired in CSOs. However, the amplitudes of a positive response-related component, reflecting error evaluation and error awareness, were strongly diminished in CSOs, even more in contact CSOs. The authors interpreted that CSOs may allocate less cognitive resources to the evaluation of committed errors, which might reflect a reduced sense of responsibility (96).

### Hormones and Neurotransmitters

The question of whether levels of the male sex hormone testosterone (T) are altered in pedophilic individuals and (child) sex offenders is rather old. This idea derived from the fact that testosterone plays a key role in all aspects of male sexuality, including sexual interest, thoughts, motivation, desire, arousal, spermatogenesis, erection, and ejaculation [for review see: (43)]. Despite this clear association between T and sexual function, the nature of this relationship remains complex, also in healthy men. One reason might be that the physiological range of testosterone levels (3–12 ng/ml or 11–40 nmol/L) is considerably higher than required for maintaining normal sexual functions. Research has also concentrated on the forensically relevant relationship between testosterone and aggression. However, as pointed out by Carré et al. (97, 98), studies on humans from as early as the seventies revealed, if at all, only weak associations between testosterone and aggression. The testosterone-behavior relationship is rather thought to be subject to individual differences and contextual variables, i.e., testosterone influences aggression especially in high dominance men, in those men with low cortisol levels, and it can affect both aggression and prosocial behavior (97, 98). In line with this, Volman et al. (70) found that psychopathic offenders exhibited less control-related anterior prefrontal activity and anterior prefrontal–amygdala coupling in a task requiring control of emotional actions, when compared with healthy control subjects. This pattern was pronounced in psychopathic individuals with high endogenous testosterone levels (70). Nowadays, it is known that, contrary to the general belief, sex offenders do not have higher testosterone concentrations than non-offenders. Recently, Wong and Gravel (99) published a carefully conducted meta-analysis, examining seven studies with a total sample size of 325 sex offenders and 196 comparison participants. They did not find a significant association between testosterone and sexual offending (99). In a current study, Kruger et al. even reported lower, but nevertheless normal testosterone levels in CSOs compared to non-offenders, which was independent of a potential pedophilic preference (54).

Even though sex offenders do not have altered testosterone concentrations, testosterone lowering treatment (TLT) has been used for 30 years to treat paraphilic patients and sex offenders. TLT leads to a profound decrease of testosterone levels, which should result in a reduction of sexual drive and, in consequence, a reduced risk of recidivism (50, 100). Typically, outcome measures comprise hormonal parameters, self-reports about sexual activity and interest or recidivism. Yet again, self-reports are susceptible to denial or faking. Hence, methods using biomarkers as “objective” measures could support treatment evaluation. To examine the effect of TLT by means of fMRI, three case studies with similar experimental designs were conducted, in which sexually relevant stimuli were presented at a supraliminal level to measure sexual interest. Based on the results of these three case studies, it can be assumed that TLT leads to a decreased activation in brain regions linked with sexual functions, especially with regard to the autonomic, the emotional, and the motivational component of sexual arousal (101–103). Results of an additional fMRI case study, in which visual sexual stimuli were presented subliminally further indicate that even at an unconscious level, TLT can lead to changes in the processing of sexually relevant stimuli, supposedly reflecting changes in cognitive and perceptive automatic processes (104). Next to fMRI, also eye tracking can be used to depict TLT induced changes. In an eye movement study with a single pedophilic CSO who was presented with pedophilic stimuli, it was found that controlled attentional processes could change under TLT whereas automatic processes remained mostly stable (104). Obviously, case studies can only provide anecdotal evidence for the usability of potential biomarkers. Nevertheless, using fMRI, EEG, or eye tracking to assess TLT induced changes in responses to sexual stimuli seems to be a promising approach for the identification of biomarkers.

According to the monoamine hypothesis, monoaminergic neuroregulatory dysfunctions are involved in the pathophysiology of paraphilic disorders (105, 106). Evidence for this comes from several sources of data. The monoamine neurotransmitters dopamine and serotonin and also norepinephrine are involved in the regulation of autonomic, motivational, and emotional sexual functions. Furthermore, they appear to modulate dimensions of human and animal psychopathology, including impulsivity, anxiety, depression, compulsivity, and pro-/antisocial behavior—dimensions that are disturbed in many paraphilic patients (106). Side effects of antidepressant (e.g., SSRIs), psychostimulant, and neuroleptic drugs in humans suggest that alterations of central monoamine neurotransmission can have substantial effects on human sexual functioning. Even though we do not fully understand the complex picture, few earlier studies indeed indicate that monoamine systems might be disturbed in paraphilic disorders [for review see: (50)]. To our knowledge, only one recent study investigated alterations in neurotransmitter levels in pedophilic sex offenders (107). By means of magnetic resonance spectroscopy, Ristow and colleagues found reduced gamma-aminobutyric acid (GABA)/Cr concentrations (γ-aminobutyric acid/creatinine) in the dorsal anterior cingulate cortex in pedophilic sex offenders (*n* = 13) compared to non-offending healthy controls (*n* = 13), which was associated with self-reported lower self-control in patients. As GABA is an inhibitory neurotransmitter, the authors interpreted the reduced GABA/cr ratio as neuronal correlate of inhibition and behavioral control in the group of pedophilic sex offenders (107).

### Penile Plethysmography

In north America, penile plethysmography (PPG) is considered the golden standard for assessing pedophilic interest. By utilizing this device, changes in penile circumference or volume in response to sexual stimuli of different ages and sexes can be assessed. A current large meta-analysis (*N* = 6,785 subjects) suggests that several phallometric testing procedures are valid indicators of pedohebephilic interest (i.e., prepubescent and pubescent children) with small to large effect sizes. Moreover, phallometric tests were able to predict sexual reoffending with moderate effect sizes (*N* = 2,709 subjects) (108). A recent large study (*N* = 1,136) showed that most sex offenders against children (83%) were unable to successfully suppress their sexual arousal to pictures depicting children when instructed to do so. Moreover, the ability to suppress sexual arousal was not associated with recidivism (109). Nevertheless, phallometric testing has been criticized for its intrusiveness, the high proportion of non-responders (probably 20–25% of all subjects), and its discriminant validity and selectivity (110). In the above mentioned study conducted by Babchishin et al., phallometric testing could only moderately discriminate between sex offenders against children and non-offending men {area under the ROC Curve (*AUC*) = 64.96%, [*CI*:.59–.69]}, a finding that did not significantly change during the suppression condition (109). However, it is likely that improving the quality of stimulus material could increase classification accuracy [up to 84.95%, (111)]. Nevertheless, PPG is recommended in DSM-5 as an additional, diagnostic marker for pedophilia (22) (see also *Ethical Considerations*).

### Eye Tracking

The earliest eye trackers were built in the late 1800s, but their relevance for sexuality has only been discovered quite recently (112). The measurement of eye movements provides the opportunity to directly explore attentional processes. Humans' ability to identify fine details is limited to two degrees of central vision, the foveal region of the retina. This limitation of acuity of the human visual system makes it possible to identify the features most interesting to the viewing subject by eye movements (113). Using eye movements, several variables can be measured, such as saccades, fixations, or pupil size [for review see: (114)]. Renaud et al. were the first to demonstrate the potential of eye tracking (and virtual-reality technology) in the assessment of deviant sexual preferences. Although there were no significant differences between pedophilic sex offenders (*n* = 8) and non-deviant controls (*n* = 8) in classical eye-movement parameters, the study provides initial indications of the potential of using eye movements in assessing pedophilic interests (115). In a later study, the same group showed in 20 healthy male subjects that eye movements can be used to assess erectile inhibition during PPG measurement (116).

Our group measured eye movements in two groups of forensic inpatients (22 pedophilic child sex offenders, 8 non-pedophilic adult sex offenders) and 52 healthy controls while they were simultaneously presented with the image of a child and an adult. Pedophiles demonstrated significantly shorter fixation latencies and significantly longer relative fixation times for child stimuli than either of the control groups. Pedophilic and non-pedophilic individuals could be classified with high sensitivity (86.4%) and specificity (90%) (117, 118). Concerning susceptibility to manipulations, a first study demonstrated that healthy hetero- and homosexual subjects (*N* = 32) could be discriminated with moderate sensitivity (77%) and specificity (86%), even though they had to mask their sexual orientation, while viewing sexual stimuli (119).

In another experimental setting, a sexual distractor task, the same subjects had to solve a cognitive task while simultaneously being presented with an image of a sexual stimulus [child or adult stimulus (120)]. Hence, this task requires attentional control in the presence of sexual stimuli. Pedophilic subjects showed significantly lower attentional control, as indicated by eye movements, than both non-pedophilic control groups. They could be discriminated with high accuracy (sensitivity: 90.9%, specificity: 84.9%). We assumed that the measured attentional control represents inhibitory executive functions, specifically interference control. Further studies will have to examine, if attentional control to sexual distractors could be linked to clinically important aspects of controllability, the capacity of self-control, and the severity of a paraphilic disorder (120). Applying the same cognitive task, outpatients with a self-reported sexual interest in children (and self-reported sexual offenses) differed from pedophilic forensic inpatients with respect to attentional control but not with regard to sexual interest (121). They demonstrated signiﬁcantly better attentional control than pedophilic forensic inpatients in the face of adult sexual stimuli, but not with respect to child sexual stimuli. This might reflect a higher capacity for self-control and self-regulation in these patients. Nevertheless, child stimuli remain highly distracting for them (121).

### Behavioral Approaches

Behavioral approaches do rather not belong to the classical biomarkers. However, as biomarkers are defined as “a characteristic that is objectively measured and evaluated as an indicator of normal biological processes, pathogenic processes or pharmacological responses to a therapeutic intervention” (1, 2), they may nevertheless be considered as such. Recently, Loth and Evans proposed the term “bio-behavioral” to refer to tests which measure fundamental social, cognitive, emotional, and motivational processes with a neurobiological basis (122). With respect to neurodevelopmental disorders, Loth and Evans discuss a possible conversion of those tests into clinically useful bio-behavioral markers. They argue that those tests can be relatively brief, cost-effective, easy to administer and interpret, and have only few risks/side effects. Furthermore, Loth and Evans argue that with respect to autism research more sophisticated biological measures [e.g., structural magnetic resonance imaging (sMRI), fMRI, EEG] do not show better discrimination accuracy between groups than cognitive/behavioral tests (e.g., emotion recognition) (122).

Like functional imaging, electrophysiological and eye tracking studies, most of the behavioral approaches aim to “objectively” assess deviant sexual interest. Similar to these measures, behavioral approaches are “i) inherently less transparent than self-report measures due to the indirect character of the measurement procedure, and ii) able to tap into automatic attitudes and behavioral dispositions, because of the implicitness of the constructs to be measured” (123). Instead of relying on self-report measures, behavioral approaches use different parameters and methods to gather information about the participant. These parameters and methods include, but are not limited to, reaction times, measurement of accuracy in a cognitive task, or evaluations of stimulus' valence and arousal. A variety of tasks are used to measure subject's responses to the presentation of sexual stimuli, e.g., evaluate valence and arousal of sexual stimuli, locate a dot on a sexually preferred or non-preferred stimulus, or name the color of a sexually preferred or non-preferred stimulus (please see below). If those approaches use response latencies during tasks to measure sexual interest, they often are called “latency-based” measures (123). Most behavioral approaches have been developed in order to assess child sexual interest, and to develop an “objective” instrument, a biomarker, which could support the diagnostic process of identifying pedophilia itself. In contrast to classical biological measures (e.g., sMRI, fMRI, EEG, hormones, neurotransmitters), behavioral approaches often have already been tested with respect to discrimination accuracy, effect sizes etc.

The viewing time (VT) approach is the most established measure of sexual interest and is based on the observation that subjects spent a larger amount of time looking at erotic than at non-erotic stimuli (124, 125). According to Schmidt and colleagues (125), VT effects can be described as prolonged decision latencies for attractiveness ratings of sexual targets. In consequence, only if participants actually followed the instructions to rate the sexual target's attractiveness, then the measure would be valid (125). Recently, Schmidt et al. conducted a large meta-analysis of VT-measures of sexual interest in children (*N* = 2,705 subjects). They concluded that VT-measures demonstrate overall moderate discriminant validity for distinguishing between sexual offenders against children and sexual offenders against adults or non-sexual offenders [*d* = .60, *CI* (.51–.69)], and significant, small to moderate convergent validity with self-reports, PPG, the Implicit Association Test (please see below), and sexual offense history measures [*r* ranging from *r* = .18 to *r = *.38 (125)]. Similarly to PPG, VT is recommended in DSM-5 as an additional diagnostic marker for pedophilia (22) (but see *Ethical Considerations*).

The Choice Reaction Time Task [CRT, (126)], and the pictorial modified Stroop task (127) rely on the concept of limited attention capacity during controlled information processing (128). Following this concept, the sexual content-induced delay (SCID), which was first proposed by Geer and Bellard (129), occurs when a salient sexual stimulus triggers attentional processes, interfering with or limiting attention to other tasks. The CRT requires the subject to locate a dot as quickly as possible while viewing sexual stimuli. According to the SCID, reaction times should be longer if dots are superimposed on images of sexually preferred stimuli compared to sexually non-preferred stimuli. In a large sample of child sex offenders and non-offenders (*N* = 233), Dombert and colleagues (130) found an overall poor to moderate effect size for the CRT (*AUC* values between .59 and .69). Additional comparisons of pedophilic and non-pedophilic sex offenders did not significantly improve the results. Interestingly, convergent validity was seen with the child sexual fantasy scale of the MSI [Multiphasic Sex Inventory (131)] (130). The pictorial modified Stroop task asks participants to name the color of stimuli while ignoring their sexual content and allows to discriminate between heterosexual and homosexual subjects (127). This notwithstanding, the task did not prove usable for distinguishing between CSOs and non-offenders. Originally developed to identify implicit racist attitudes, the Implicit Association Test [IAT (132) was adapted to measure implicit child-sex associations to discriminate pedophiles from non-pedophiles]. In a meta-analysis Babchishin et al. (133) analyzed 12 distinct samples of child sex offenders and different control groups (*N* = 707), in order to examine discriminant and convergent validity of IAT-measures to assess sexual interest in children. They found that IAT measures were able to distinguish between CSOs and non-offenders with large effect size [mean weighted *d* = .96, *CI* (.67–1.24)]. Effect sizes decreased when comparison groups were non-sex offenders or rapists (*d* = .58 resp. *d* = .48). Convergent validity was supported by correlations with VT-measures in few studies, self-report, and sexual offense history variables (*r* ranging from *r* = .27 to *r* = .30) (133). In an adapted IAT, the go/no-go association task (GNAT, a combination of the IAT and the go/no-go response inhibition task) Bartels and colleagues (134) could distinguish between individuals with a history of exclusive extrafamilial sexual offenses against children and those with exclusive intrafamilial offenses or offenses against both children and adults (*N* = 70) with good discrimination accuracy (*AUC* = .71, p < .007). However, only partial support was found for convergent validity between the GNAT and questionnaires on sexual thoughts and fantasies (134).

Those single measures of sexual interest are promising, but effect sizes are only small to moderate. Therefore, multimodal approaches have been proposed in order to develop a clinically applicable tool for the assessment of sexual interest with high reliability and validity. Banse and colleagues (135), for instance, introduced the Explicit and Implicit Sexual Interest Profile (EISIP), which combines direct self-report and indirect latency-based measures, i.e., IAT and VT. In a first study, the EISIP demonstrated an overall high discriminative validity (*AUC* = .95, *p* < .001) between child sex offenders and controls (non-sex offenders, non-offenders, whole sample size *N* = 113 subjects) (135). In a similar study Babchishin et al. could partially replicate these results, with significant differences between the groups regarding VT and self-report but not with respect to an IAT (136). The authors argued that besides the relatively small sample size (*N* = 56), a gender-related IAT probably would yield better results than the neutral one that they had used in the study. Recently, several latency-based measures of sexual interest (VT measures, CRT, pictorial modified Stroop task, modified IAT) were combined with psychophysiological measures, i.e., pupillary responses, and sexual fantasy questionnaires, in order to assess sexual interest in 102 community men (137). Correlations between measures were positive, albeit effect sizes varied from small to large. Additionally, convergent and concurrent validity was shown for latency-based measures and pupil-dilation. Combined indices of sexual preference for adult stimuli predicted 75% of the variance in self-reported sexual interest in adults (137).

Applying a completely different approach, Fromberger et al. conducted a pilot study to test if behavioral monitoring of CSOs in high-immersive virtual risk situations provides additional information for risk management (138). Six pedophilic CSOs and seven non-offender controls walked through three virtual risk situations (supermarket), where they were confronted with a virtual child character. In 89% of cases, CSOs showed behaviors not in line with their own beliefs about adequate behavior in comparable risk situations. Although this was only a pilot study, results suggest that virtual risk scenarios could provide practitioners with the opportunity to monitor the behavior of CSOs, and to test their decisions on unsupervised privileges without endangering the community. This may provide additional information for therapy progress (138).

## Are There Any Biomarkers for Pedophilia and Child Sexual Abuse?

**Figure 1:**
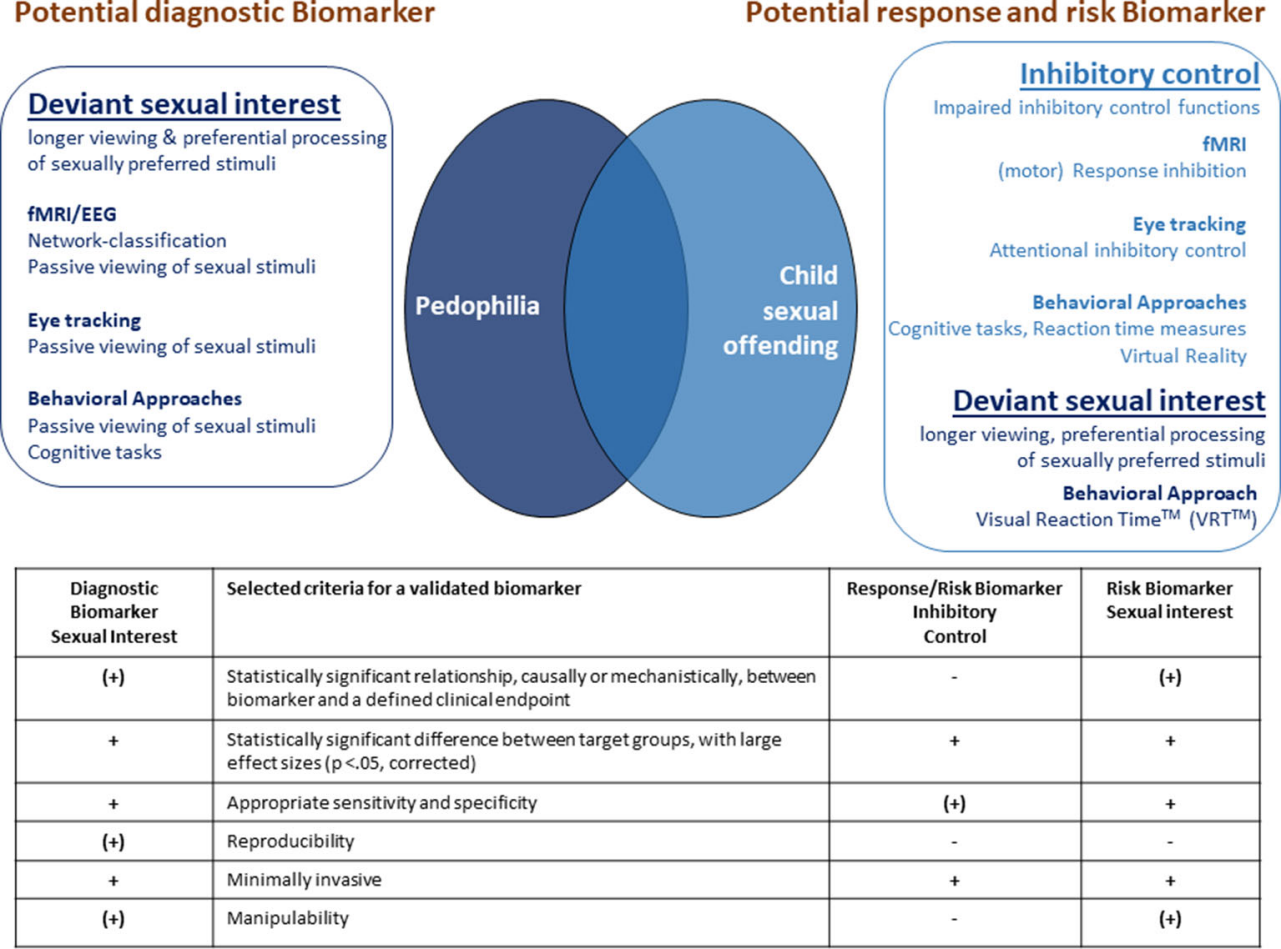
Suggested potential composite diagnostic, response and risk biomarkers for pedophilia and child sexual offending. The table summarizes the evaluation of these potential biomarkers according to selected criteria for validated biomarkers. +: fulfills the criterion, (+): fulfills the criterion with restrictions, -: does not fulfill the criterion.

In the following, we will discuss and evaluate the above described biological, psychophysiological, and behavioral findings with respect to their usefulness as clinically important biomarkers for pedophilia and/or child sexual offending (see **Figure 1**). Thereby, if possible, we will refer to the scheme proposed by Prata et al. to evaluate biomarkers in psychiatry [see above, (4)]. According to these authors, valid biomarkers fulfill the following criteria: i) their relationship to a certain clinical endpoint is reliable (statistical significant), ii) plausible (causally or mechanistically understandable), iii) accurate (sensitive and specific), and iv) reproducible across clinically relevant settings. Moreover, even valid, clinically applicable biomarkers have to provide a meaningful advantage into decision-making or clinical care.

### Potential Diagnostic Biomarkers to Assess Sexual Interest in Children

According to Califf (139), diagnostic biomarkers detect or confirm the presence of a disease or condition of interest, or identify an individual with a subtype of the disease. Susceptibility/risk biomarkers, on the other hand, can indicate the potential for developing a disease or medical condition in an individual who does not currently have the clinically apparent disease or medical condition [for more details see: (1, 2, 139)].

#### Functional Magnetic Resonance Imaging, Penile Plethysmography, Eye Tracking, and Behavioral Approaches to Assess Sexual Preference

As seen above, the assessment of deviant sexual interest using fMRI, PPG, eye tracking, or behavioral approaches has a long tradition in the forensic research context (see *Structural and Functional Imaging*, *Penile Plethysmography*, *Eye Tracking*, *Behavioral Approaches*). In comparison to other parameters, these approaches are most likely to fulfill criteria for a validated and clinically meaningful diagnostic biomarker to assess sexual interest in children (see **Figure 1**).

Applying fMRI, pedophilic and non-pedophilic subjects could be classified with high sensitivity and specificity while viewing sexually preferred stimuli (86, 87). Even though independent replication studies examining reliability and validity in a sample of non-admitting subjects are necessary, this approach is a promising tool to support the assessment of deviant sexual interest in the diagnostic process. Furthermore, susceptibility to manipulation has to be examined.

Also, PPG seems to be a valid indicator for pedohebephilic interest, and is able to predict sexual recidivism (108). However, discrimination accuracy is only moderate, the number of non-responders seems to be high, and ethical issues regarding the intrusiveness of this measure should not be underestimated (109, 110). The measurement of eye movements while being presented with sexually relevant and sexually non-relevant stimuli seems to be a promising, potential tool to discriminate between pedophilic sex offenders and non-pedophilic controls (offenders and non-offenders) with high accuracy (117). However, independent replication studies are necessary and reliability and validity remain to be assessed. Furthermore, susceptibility to manipulations has to be examined with the target groups. Besides PPG, also behavioral measures of sexual interest are well studied, especially with respect to psychometric criteria such as reliability, validity, and discrimination accuracy. They show small to moderate discrimination accuracy between pedophilic and non-pedophilic subjects, and between CSOs and controls. However, as aforementioned, convergent validity was not always tested, and effects were small. Multimodal approaches have shown that combining self-reports, latency-based measures, and psychophysiological measures could enhance reliability, validity, and discrimination accuracy (135–137). Although those multimodal approaches have to be replicated and tested for susceptibility to manipulation in large groups of pedophilic and non-pedophilic men with and without sexual offenses as well as healthy controls, they provide a first step in the development of the aforementioned “composite biomarkers,” i.e., composite measures of several parameters. Recently, Demidowa and colleagues recommended a more intensive international collaboration of sexual behavior assessment labs in order to establish the most effective methods for the assessment of sexual interest, and to facilitate standardization and evaluation procedures (140). It would be of interest, if the combination of different approaches (e.g., ESIP, eye tracking-, and fMRI-measures) will carry forward the development of a composite biomarker to assess deviant sexual interest.

Considering the aforementioned criteria for a validated biomarker, one critical point remains: the relationship between this potential diagnostic biomarker and a defined clinical endpoint. A “sexual interest biomarker” can assess sexual interest at that moment. However this measured sexual interest in children obviously should not be equated with a diagnosis of pedophilic sexual orientation according to DSM-5, because it does not deliver any information on the continuity of this preference, or any fantasies and sexual urges. Furthermore, a clinical diagnosis of pedophilia according to ICD-10, and pedophilic disorder according to DSM-5 requires feelings of guilt, shame, or anxiety about these impulses or interpersonal difficulty or behaviors involving sexual activity (21, 22). Hence, a “sexual interest biomarker” could provide clinicians with supportive information on a sexual interest in children, especially with regard to social desirability, denial, or faking attempts. It has a significant relationship to the clinical endpoint (i.e. a diagnose). This notwithstanding, in our view, a “sexual interest biomarker” cannot be equated with a diagnosis of pedophilia/pedophilic sexual orientation or pedophilic disorder.

### Potential Biomarkers for Therapy Response and Risk Assessment

To evaluate therapy or assess risk of recidivism, several types of biomarkers can be used. Generally, pharmacodynamic/response biomarkers change in response to exposure to a medical product or an environmental agent. Changes in or the presence of predictive biomarkers predict that an individual or a group of individuals is more likely to experience a favorable or unfavorable effect from exposure to a medical product or environmental agent. Prognostic biomarkers, on the other hand, identify the likelihood of a clinical event, disease recurrence, or disease progression in patients with a disease or medical condition of interest [for more details see: 1, 2, 139)]. In the following, we will therefore discuss the usability of the methods and findings discussed above as biomarkers in therapy and risk assessment.

#### Functional Magnetic Resonance Imaging, Eye Tracking, and Behavioral Approaches to Assess Inhibitory Control in Therapy and Risk Assessment

In addition to the assessment of deviant sexual interest, fMRI, eye tracking, and behavioral methods have been used to characterize cognitive functioning in pedophilic subjects and CSOs (see *Structural and Functional Imaging*, *Eye Tracking*, *Behavioral Approaches*). Inhibitory control functions are of special interest with respect to therapy and risk assessment in those groups. Sex offender treatment programs rely on cognitive or behavioral interventions to reduce the risk of recidivism. Cognitive behavioral therapies are intended to change internal processes—thoughts, beliefs, emotions, physiological arousal—alongside changing overt behavior, such as social skills or coping behavior (141). Control functions, as a part of executive functions, are of importance for those behaviors. In treatment, offenders learn to monitor and control thoughts, feelings, and behaviors associated with offending, in order to adopt alternative ways of coping with deviant sexual thoughts and desires. Moreover, general self-regulation problems belong to psychologically meaningful risk factors of sexual recidivism (142).

Results of fMRI, and neuropsychological studies found impaired (motor) response inhibition functions in sexual offenders against children (90, 92). Recent studies revealed that a worsened response inhibition ability is related to offense status rather than pedophilic preference (91, 93).

Supporting these results, functional imaging studies have shown that a diminished fronto-limbic functional connectivity at resting state is rather linked to child sexual offending than to pedophilia (67, 68). As discussed in *Structural and Functional Imaging*, a diminished fronto-limbic functional connectivity could be seen as a neurobiological correlate for an (generally) impaired ability to exert inhibitory control over behavior, which seems to be associated with child sexual offending. Furthermore, pedophilic CSOs also seem to have lower attentional control capacities than non-pedophilic controls, as was shown in a sexual distractor task with high discrimination accuary while measuring eye movements (120). Finally, the application of virtual reality approaches could be promising to examine a more realistic, inhibitory control behavior; for instance when confronting participants with children in a virtual supermarket (138).

In conclusion, these studies suggest that inhibitory control functions at simple motoric, attentional, as well as at complex behavioral level could be related to (pedophilic) child sexual offending. According to the above mentioned significance of inhibitory control functions in therapy, they potentially also might be of interest in therapy evaluation. Hence, the assessment of inhibitory control functions seems to be an interesting potential biomarker for therapy and risk assessment. However, currently none of the above discussed approaches are tested for reliability, validity, and susceptibility for manipulation. Furthermore, it is not clear if these parameters are specific for child sexual offending. A diminished functional fronto-limbic connectivity, for instance, is also seen in violent and psychopathic offenders. Therefore, it might be a potential biomarker within the groups of subjects with a sexual interest in children, to “indicate” the risk of offending, but not a general “screening” biomarker. Additionally, the four structural and functional imaging studies derive from the same research collaboration (NeMUP, www.nemup.de), hence they will have to be replicated by independent research groups.

#### Behavioral Approaches for Risk Assessment

To the best of our knowledge, Gray and colleagues published the only study applying a behavioral approach to predict sexual recidivism in CSOs (143). They investigated the applicability of the so called Visual Reaction Time™ (VRT™), a version of the aforementioned viewing time (VT) approach. Gray and colleagues were interested to measure sexual interest in children in order to predict sexual recidivism among men who sexually abused children and men with other sexually deviant behaviors (*N* = 621). VRT™ to children was significantly related to sexual recidivism over a 15-year period, and independent of the specific deviant sexual interest. The VRT™ showed a moderate to large effect size for differentiation between re-offenders and non-re-offenders (Cohen's *d* = .71). When dividing participants into three groups based on their VRT™, the 97 participants with a VRT™ lower than one standard deviation (*SD*) below mean had not relapsed. Relapse rates in the 432 subjects with a mean VRT™ and the 92 participants with a VRT™ higher than one *SD* above the mean, on the other hand, were higher and amounted to 7 and 27%, respectively (143). An important limitation of this study was that only 22 out of the 621 subjects were registered as re-offenders. The VRT™ is a promising approach to become a risk biomarker, however, replication studies with larger samples of child sexual abusers are necessary to confirm the results. The authors also proposed to evaluate, if VRT™ scores can be used to monitor treatment progress. That is, it should be examined if treatment can lower an abuser's VRT™ to children and if this reduction correlates with risk of recidivism. If VRT™ indeed allows conclusions about treatment progress and recidivism risk, it could further be incorporated into existing actuarial recidivism indices (143).

### Neurobiological Findings for Pedophilia and Sexual Offending—Currently Not Suitable to Serve as Potential Biomarkers

In our view, the findings summarized below are useful to characterize pedophilia and child sexual offending and enhance our knowledge about neurobiological underpinnings. Potentially, they could help to better discriminate between pedophilia and child sexual offending. However, at this point in time, we believe that it is still unclear if they could provide potentially clinically applicable biomarkers.

#### Prenatal Factors

The above discussed research (see: *Genetic and Prenatal Factors*) indicates that genetic and prenatal factors, such as birth stress, epigenetic modifications, or prenatal testosterone, potentially leading to MPAs, congenital malformations and non-right handedness, are small but relevant risk factors for the development of pedophilia and/or the occurrence of child sexual offending. Sample sizes in the reported studies are large, and effect sizes vary from small to moderate. However, up to date, we do not certainly know if those factors are rather associated with pedophilia, child sexual offending, or any sexual, or non-sexual offending. One approach to get more clarification could be to split groups more sophisticatedly regarding sexual orientation, that is, to include variables such as preferred gender and age, and also offense status in the analyses (54, 144). Additionally, genetic effects are small, and did not survive corrections for multiple testing (25). Furthermore, it is known that most of the MPAs are not unique to pedophilia, autism, schizophrenia, attention deficit hyperactivity disorder, or any other psychiatric disorder (145). To our knowledge, the question of which circumstances may result in which specific psychopathological signs is still under debate. Moreover, the frequency of the pre- and perinatal anomalies examined is rather low. Babchishin et al., for instance, reported any congenital malformation in 47 out of 654 offenders (7.2%) (35). Fazio et al. pointed out that, even though they found a significant relationship between handedness and pedophilia, handedness accounted for only about 0.2% of the variance in pedophilia (39). However, one has to keep in mind that, considering the low base rate of pre- and perinatal anomalies, their explanatory power is rather limited.

#### Hormones and Neurotransmitters

Based on the current knowledge (see *Hormones and Neurotransmitters*), the actual basal testosterone concentration could probably not serve as a biomarker for child sexual offending or pedophilia. Regarding the complex relationship between testosterone, sexuality, aggression, and also individual and context characteristics, a multimodal assessment could help to disentangle this network [for instance see: (70)]. If fMRI-approaches are suitable to evaluate TLT, has to be shown in the future.

Furthermore, up to date, we do not fully understand the complex picture with respect to neurotransmitter changes in pedophilia and child sexual offending [for a review see: (50)]. Besides the study by Ristow et al. (107), further studies connecting directly measured neurotransmitters with other biological, behavioral, or criminological parameters remain to be conducted.

#### Structural Imaging

As mentioned in *Structural and Functional Imaging*, altered brain structures might rather be associated with child sexual offending than with pedophilia (64, 65). Nevertheless, structural brain alterations are well known for several psychiatric diseases. Although the effects in offending pedophiles seem to be specific, reliability and validity has not yet been evaluated. Classification analyses to discriminate different patient groups, an approach that is already being used in the case of other psychiatric disorders, are necessary. For instance, there is evidence that it is possible to distinguish cortical abnormalities in bipolar disorder from schizophrenia by means of machine learning (13). Future research will have to examine if approaches like this could also prove useful in the classification of CSOs.

#### Functional Imaging—Electroencephalography

Even though the two EEG-studies examining sexual interest and response inhibition respectively yielded interesting results, they have to be replicated with larger, well-defined groups. Discrimination accuracy has to be shown, as well as reliability and validity (95, 96).

### Ethical Considerations

Public, clinical, and scientific expectations regarding biomarkers are particularly high. Hence, it is possible that any clinical application of biomarkers would be quickly implemented, without time for reflections on social and ethical issues (146).

The worldwide accepted principles of biomedical ethics by Beauchamp and Childress comprise i) respect for autonomy (a norm of respecting and supporting autonomous decisions), ii) nonmaleficence (a norm of avoiding the causation of harm), iii) beneficence (a group of norms pertaining to relieving, lessening, or preventing harm and providing benefits and balancing benefits against risk and costs), and iv) justice [a group of norms for fairly distributing benefits, risks, and cost (147)]. These principles should be equally applied in biomarker research and application. With respect to the topic of this review, the distinction between a measured sexual interest in children (e.g., using a biomarker) and a diagnose of pedophilia/pedophilic disorder is of great significance. It is a common public misconception that all people with pedophilia/pedophilic disorder have, or will sexually abuse a child (148). Hence, pedophilia/pedophilic disorder is among the disorders that lead to serious stigmatization and social rejection (149, 150). Bearing this in mind, a “sexual interest biomarker” potentially could be considered as a “biomarker for pedophilia” by the general public, and may raise the expectation of an “objective, infallible” screening marker for pedophilia and child sexual abuse. Therefore, we should be careful regarding the significance of a “biomarker to assess sexual interest in children.” Similarly, the VRT™ approach to asses sexual recidivism should be applied with precaution, and obviously only as an additional tool, potentially supporting established risk assessment instruments and clinical evaluation. Labeling a person with a strong pedophilic VRT™ score as a person of high risk of relapse based only on this score is a serious stigmatization. The same holds true for a potential response and risk biomarker which assesses inhibitory control functions. To classify a person with poor inhibitory control functions as a person with high recidivism risk and low therapeutic success, based solely on this marker, clearly is stigmatizing and unethical.

These short considerations point to special ethical challenges which arise with the development of potential biomarkers for pedophilia and child sexual abuse.

## Conclusion

The objective of this review was to examine the literature regarding potential biomarkers of pedophilia and child sexual offending and to describe their usefulness with respect to the diagnostic process, treatment evaluation, and risk assessment. First we presented an overview of the current neurobiological knowledge, as well as physiological and psychophysiological approaches to characterize pedophilia and child sexual offending. Secondly, we discussed and evaluated the impact of this knowledge on the development of biomarkers for diagnosis, therapy response, and risk assessment in pedophilic subjects and CSOs.

The development of a composite diagnostic biomarker to assess deviant sexual interest, combining several measures like fMRI, eye tracking, and behavioral approaches (including VR) seems to be most promising. Probably, characterizing sexual interest at different information processing levels (electrophysiological and hemodynamic responses, behavioral, and attentional processes) could lead to synergetic effects, thereby enhancing discrimination accuracy. A valid and reliable measurement of deviant sexual interest, insensitive to manipulations, could significantly support the clinical diagnostic process. With respect to pedophilia, such a biomarker would be a specific instrument, as it would aim to measure one of the core signs of pedophilia, that is, a sexual interest in children. Nevertheless, the underlying principles of this method, i.e., the measurement of attentional processes toward individually salient stimuli/objects, is not specifically bounded to the assessment of a deviant sexual interest. Rather, it could also be transferred to other diagnostic settings. Similarly, regarding therapy evaluation and risk assessment, a composite biomarker to assess inhibitory control functions seems to be promising. This should be accomplished at different information processing levels, but also with respect to different inhibitory control functions, such as simple (motor) response inhibition, cognitive inhibitory control, and complex behavioral control functions. In contrast to the biomarker of sexual interest, this potential biomarker would not be a specific marker for pedophilia and/or sexual child abuse. As mentioned above, impaired inhibitory control functions are known also for violent and psychopathic offenders, and their improvement is a central part of offender treatment in general.

In conclusion, a lot of research has enhanced our neurobiological knowledge about pedophilia and child sexual offending. However, in our view, according the criteria by Prata and colleagues (4), currently none of the above discussed parameters and approaches is ready to serve as a clinically applicable diagnostic, response, or predictive biomarker for pedophilia and child sexual offending. Therefore, further work remains to be done in order to identify the most useful biomarkers.

The application of the so called Research Domain Criteria (RDoC), a research initiative for new approaches to investigating and classifying mental disorders, made an approach to carry forward the research (accessed 18th June 2019, https://www.nimh.nih.gov/research/research-funded-by-nimh/rdoc/index.shtml). Abi-Dargham et al. suggested that this approach is directly relevant to the search for biomarkers because it aims to identify valid elements, such as genes, molecules, cells, circuits, physiological measures, or behavior, that are associated with specific cognitive constructs across different systems (5). Altered inhibitory control functions, for instance, are a common deficit in several mental disorders, and not limited to any DSM-category or child sexual offending in particular. The RDoC approach contains a domain “cognitive systems” including the construct “cognitive control” (accessed 18^th^ June 2019, https://www.nimh.nih.gov/research/research-funded-by-nimh/rdoc/constructs/cognitive-systems.shtml). Bringing together research results about impaired cognitive control, independent of any diagnosis, would enhance our knowledge about this construct and alterations in mental disorders, and hence facilitate the development of appropriate biomarkers.

## Author Contributions

KJ conducted literature searches and wrote the first draft of the manuscript. TW gave substantial linguistic support. TW, PF, IM and JM were involved in an intensive drafting and revision of the manuscript. All authors have read and approved the final manuscript.

## Conflict of Interest

The authors declare that the research was conducted in the absence of any commercial or financial relationships that could be construed as a potential conflict of interest.
